# Under-recording of diabetes on medical record may be associated with adverse in-hospital outcomes

**DOI:** 10.1186/1758-5996-7-S1-A96

**Published:** 2015-11-11

**Authors:** Rogerio Silicani Ribeiro, Marcia Nery, Jose Antonio Maluf de Carvalho, Ana Claudia Latronico, Magda Tiemi Yamamoto, Thalita Barrera Modena, Adriana de Fatima Avansi

**Affiliations:** 1 Universidade Federal de São Paulo, São Paulo, Brazil

## Background

Hospitalized patients with diabetes (DM) and stress hyperglycemia (SH) have higher risk of complications and mortality compared to normal (NL), especially if hypo or hyperglycemia come about. The under-recording of diabetes may difficult the recognition of glucose abnormalities, delay adequate therapy and aggravate prognosis of non-registered diabetics (DMNR).

## Objective

To compare in-hospital outcomes among patients with DMNR, DM, SH and NL.

## Materials and methods

This is a retrospective analysis including 62136 adults (> 18 yrs.) admissions in groups NL (69%), DM (17%), DMNR (5%), and SH (9%), hospitalized between 2010 and 2013. Patients were classified according to five coded discharge diagnoses registered in electronic medical record. The inclusion criteria for DMNR were registration of DM in previous admissions and omission of diagnosis in consecutive readmission. Inclusion criteria were length of stay between 2 and 120 days, availability of capillary glucose (at least one measurement for NL patients and two measurements for DM, DMNR and SH patients). Exclusion criteria were pregnancy. In hospital outcomes included in the retrospective analysis were nosocomial infection, sepsis, intensive care unit admission and death. To compare the proportion of adverse outcomes we perform chi-square test.

## Results

Average age was 54,8 yrs., the proportion of females was 52%. The proportion of hospitalizations with surgical interventions was 44%. The average length of stay among NL, DM, DMNR and SH, were 4,7; 7,3; 13,9 and 17,1 days respectively. The incidence of nosocomial infection were 0,4%; 0,9%; 2,3% and 3,9% and the incidence of sepsis were 1,5%; 4.0%; 13,7% and 16% in NL, DM, DMNR and SH, respectively. The mortality rate were 0,8%, 1,4%, 10,2% and 12,7%, respectively. P values were < 0.05 for every outcome described.

## Conclusion

The omission of the diagnosis of DM on medical record is associated with adverse in-hospital outcomes.

